# Identification of a dysfunctional microglial population in human Alzheimer’s disease cortex using novel single-cell histology image analysis

**DOI:** 10.1186/s40478-020-01047-9

**Published:** 2020-10-20

**Authors:** Molly E. V. Swanson, Emma L. Scotter, Leon C. D. Smyth, Helen C. Murray, Brigid Ryan, Clinton Turner, Richard L. M. Faull, Mike Dragunow, Maurice A. Curtis

**Affiliations:** 1 Department of Anatomy and Medical Imaging, Faculty of Medical and Health Science, University of Auckland, Private Bag 92019, Auckland, New Zealand; 2 Centre for Brain Research, Faculty of Medical and Health Science, University of Auckland, Private Bag 92019, Auckland, New Zealand; 3 School of Biological Sciences, Faculty of Science, University of Auckland, Private Bag 92019, Auckland, New Zealand; 4 Department of Pharmacology and Clinical Pharmacology, Faculty of Medical and Health Science, University of Auckland, Private Bag 92019, Auckland, New Zealand; 5 Department of Anatomical Pathology, LabPlus, Auckland City Hospital, Auckland, New Zealand; 6 Present Address: School of Biological Sciences, Faculty of Science, University of Auckland, Private Bag 92019, Auckland, New Zealand; 7 Present Address: Department of Pathology and Biomedical Science, Centre for Free Radical Research, University of Otago, 2 Riccarton Avenue, Christchurch, 8011 New Zealand

**Keywords:** Microglia, Alzheimer’s disease, Tau, Amyloid beta, Dysfunction, Immunohistochemistry, Single-cell analysis

## Abstract

In Alzheimer’s disease (AD), microglia are affected by disease processes, but may also drive pathogenesis. AD pathology-associated microglial populations have been identified with single-cell RNA-Seq, but have not been validated in human brain tissue with anatomical context. Here, we quantified myeloid cell markers to identify changes in AD pathology-associated microglial populations. We performed fluorescent immunohistochemistry on normal (n = 8) and AD (n = 8) middle temporal gyri, co-labelling the pan-myeloid cell marker, Iba1, with one of 11 markers of interest (MOIs): CD45, HLA-DR, CD14, CD74, CD33, CD206, CD32, CD163, P2RY12, TMEM119, L-Ferritin. Novel image analyses quantified the single-cell abundance of Iba1 and each MOI. Each cell was gated into one Iba1-MOI population (Iba1^low^ MOI^high^, Iba1^high^ MOI^high^, or Iba1^high^ MOI^low^) and the abundance of each population was compared between AD and control. Triple-labelling of L-Ferritin and Iba1 with a subset of MOIs was performed to investigate L-Ferritin-MOI co-expression on Iba1^low^ cells. Iba1^low^ MOI^high^ myeloid cell populations delineated by MOIs CD45, HLA-DR, CD14, CD74, CD33, CD32, and L-Ferritin were increased in AD. Further investigation of the Iba1^low^ MOI^high^ populations revealed that their abundances correlated with tau, but not amyloid beta, load in AD. The Iba1^low^ microglial population highly expressed L-Ferritin, reflecting microglial dysfunction. The L-Ferritin^high^ CD74^high^ HLA-DR^high^ phenotype of the Iba1^low^ population mirrors that of a human AD pathology-associated microglial subpopulation previously identified using single-cell RNA-Seq. Our high-throughput immunohistochemical data with anatomical context support the microglial dysfunction hypothesis of AD.

**Electronic supplementary material:**

The online version of this article (10.1186/s40478-020-01047-9) contains supplementary material, which is available to authorized users.

## Background

Alzheimer’s disease (AD) pathology is characterised by the accumulation of amyloid beta plaques and hyperphosphorylated tau tangles [1]. The aggregation of amyloid beta proteins precipitates inflammatory reactions by microglia, the innate immune cells of the central nervous system [2, 3]. Genetic risk factors for AD include loss-of-function mutations in microglia-specific genes, including *CD33* and *TREM2*, which are associated with reduced microglial phagocytic capacity [4–7]. Such microglial-specific genetic risk factors for AD suggest that microglial reactions are not only a result of disease processes, but may drive disease pathogenesis.

Changes in microglial function may impact the development of AD through different processes. The pro-inflammatory microglia hypothesis that posits that deposition of amyloid beta and subsequent phagocytosis by microglia drive chronic microglial activation, making them ‘neurotoxic’ [8, 9]. Alternatively, the microglial dysfunction hypothesis proposes that AD pathogenesis is modified by loss of normal microglial function, encompassing both dysregulated microglial activation and loss of microglial ‘neurosupportive’ function [10–14]. With two disparate hypotheses, it is unclear whether microglial activity should be enhanced or reduced in order to reduce AD pathology, although it is also possible that both hypotheses are correct but reflect different stages of progression.

Indeed, the traditional dogma of microglia existing in either a pro-inflammatory ‘neurotoxic’ or anti-inflammatory ‘neurosupportive’ state in the human brain oversimplifies the heterogeneity of microglial reactions [15]. Microglial heterogeneity in the normal and diseased brain is now being characterised using novel technologies, including single-cell and single-nuclei RNA sequencing [16–19]. AD pathology-associated microglial subpopulations with unique transcriptomic signatures have been identified, including a population with enriched expression of *FTL* (L-Ferritin), *HLA*-*DRA*, *HLA*-*DRB1*, *CD14*, and *CD74* [16, 17] (Additional file 1: Table S1). However, these studies require the dissociation of cells from whole tissue. As such, they are unable to resolve whether changes in microglial populations are driven by human AD pathology or quantify the anatomical location of microglia with unique expression profiles relative to amyloid beta and tau in the brain.

By contrast, immunohistochemical studies in the post-mortem human brain have identified significant changes in the expression of single proteins in microglia associated with amyloid and tau pathology, including HLA-DR, CD45, CD14, CD32, CD163, and L-Ferritin, which are proposed to alter microglial function and influence disease pathogenesis [6, 16, 17, 20–51] (Additional file 1: Table S1). Despite growing evidence linking microglial changes to AD pathogenesis, a recent systematic review revealed marked differences in microglial protein changes in post-mortem human AD tissue between studies (for review see Hopperton et al. [48]). The most consistent changes identified include increases in classical ‘activation’ proteins, like HLA-DR and CD68, while numerous other proteins with more specific functions show high variability between studies or are infrequently assessed [48].

A key contributor to this variability between immunohistochemical studies is the methodology employed to quantify microglial protein abundance. In this study, we developed a novel single-cell image analysis pipeline to investigate multiple myeloid cell markers previously identified as altered in the human AD brain or enriched in an AD pathology-associated microglial population. The markers included proteins more highly expressed by, or specifically expressed by, perivascular macrophages. Because all myeloid cell markers selected for investigation in this study have significance to AD, we have collectively termed them markers of interest (MOI). The advantages of our approach are identification of multiple key microglial proteins, quantification of their abundance at the single cell level, and retaining anatomical context allowing correlation of marker abundance in proximity to amyloid and tau.

Specifically, we investigated the single cell abundance of eleven MOIs (Additional file 1: Table S1) across microglial and perivascular macrophage populations in immunohistochemically stained normal and AD cortex; CD45, HLA-DR, CD14, CD74, CD33, CD206, CD32, CD163, P2RY12, TMEM119, and L-Ferritin. Changes in protein expression were quantified at the single cell level and reflect not only any overall microglial changes in AD, but changes in specific populations associated with the AD pathology markers, amyloid beta, and tau. This work demonstrates a clearer picture of multiple microglial population changes in AD human brain.

## Methods

### Human tissue selection

Formalin-fixed middle temporal gyrus (MTG) blocks from eight neurologically normal and eight AD cases from the Neurological Foundation Human Brain Bank were used in this study (Table 1). The AD cases had a history of dementia and were considered at least intermediate AD, based on their National Institute of Aging-Alzheimer’s Association (NIA-AA) ‘ABC’ score. Cases with Lewy body disease or other neurodegenerative pathology were excluded. Normal cases had no previous history of neurological disorders and cause of death was unrelated to any neurological condition. An initial independent pathology assessment reported no other disease pathology aside from normal age-related amyloid presence. The eight normal cases were included in the analysis based on this first pathology assessment. A more comprehensive pathology assessment, including determination of the ‘ABC’ scores, was carried out on all normal cases following our analysis. Of the eight normal cases, seven showed no or low AD neuropathologic changes. However, one normal case (H187) showed an intermediate AD neuropathologic change despite the initial pathology report suggesting pathological changes were in the normal range for its age. Given the changes were not high AD changes, the relatively older age of this case, and the lack of neurological symptoms at time of death, we have maintained the normal classification of this case for our analysis.

**Table 1:** Human cases used for this study

Case	Age (years)	Sex	Post-mortem delay (h)	NIA-AA staging (AD neuropathologic change)
*Normal*				
H169	81	Male	24	A2 B0 C1 (none)
H180	73	Male	33	A2 B0 C1 (none)
H187	98	Female	15	A2 B2 C1 (intermediate)
H191	77	Male	25	A0 B0 C0 (none)
H196	85	Male	15	A1 B0 C0 (none)
H229	88	Female	17	A2 B1 C2 (low)
H243	77	Female	13	A0 B0 C0 (none)
H246	89	Male	17	A0 B1 C0 (none)
Mean	83.5 ± 9.1	–	19.3 ± 6.5	–
*AD*				
AZ99	94	Female	8.5	A3 B3 C2 (high)
AZ102	84	Female	14.5	A3 B2 C2 (intermediate)
AZ107	86	Male	N/A	A2 B3 C1 (intermediate)
AZ108	94	Female	11.5	A3 B3 C2 (high)
AZ109	90	Female	31	A3 B2 C1 (intermediate)
AZ110	86	Female	15	A3 B3 C2 (high)
AZ113	77	Male	3.5	A3 B3 C2 (high)
AZ119	89	Male	5	A3 B2 C2 (intermediate)
Mean	87.5 ± 5.6	–	12.7 ± 9.2	–

### Free-floating immunohistochemistry

All MOIs investigated in this study were co-labelled with the pan-myeloid cell marker, Iba1, ensuring that functional changes across microglial and perivascular macrophage populations were assessed. As part of the Neurological Foundation Human Brain Bank procedures, the MTG is cut into 4 blocks, of which we used one for this study; typically block 1 or 2 to ensure we were sampling from the middle of the MTG in all cases [52]. For Iba1 co-labelling with MOIs, three 50-μm thick MTG sections approximately 800 μm apart were selected. Free-floating fluorescent immunohistochemistry was performed as previously described [53, 54, 57]. Sections were incubated in Tris–EDTA pH 9.0 for antigen retrieval, and subsequently in primary antibodies against Iba1 and an immunophenotype MOI (Additional file 1: Table S2). Iba1 was visualised using AlexaFluor^®^ 594- or 647-conjugated secondary antibody and immunophenotype MOIs were visualised using AlexaFluor^®^ 488- or 594-conjugated secondary antibody or the AlexaFluor^®^ 488 TSA protocol (Additional file 1: Table S2). For AD pathology load analysis, two MTG sections approximately 800 μm apart were selected per pathology stain. Formic acid antigen retrieval was performed to unmask aggregate epitopes. Sections were incubated in anti-amyloid beta and anti-tau antibodies, visualised using species-specific AlexaFluor^®^-conjugated secondary antibodies. Nuclei were counterstained with Hoechst. Sections were mounted using PBS and coverslipped using ProLong^®^ Diamond Antifade mounting media.

For representative images demonstrating each MOI co-labelling with Iba1, sections were imaged on an Olympus FV1000 confocal microscope (60× oil). Optical *z*-stacks were taken through the entirety of the cell body and processes. For quantification, MTG grey matter images were acquired for each section using a Nikon Ni-E microscope (20×, 0.5 NA) with Nikon DS-Ri2 and Qi2 cameras and a motorised stage. Regions of interest (ROIs) encompassing grey matter layers I–VI were imaged based on the Hoechst counterstain. These images were used for (1) the tissue-wide integrated intensity analysis, (2) the single-cell Iba1-MOI analysis for both Iba1-MOI and Iba1-L-Ferritin-MOI co-labelling, and (3) the AD pathology load analysis.

### Paraffin immunohistochemistry

For the amyloid beta spatial analysis, two sequential 10-μm thick MTG sections were selected from AD cases and received two immunohistochemical staining rounds; Iba1/L-Ferritin/HLA-DR, then amyloid beta/tau. Paraffin immunohistochemistry was performed as previously described [58]. Tris–EDTA pH 9.0 antigen retrieval was performed, and sections incubated in primary then AlexaFluor^®^-conjugated secondary antibody mixtures. Nuclei were counterstained with Hoechst. Sections were mounted using PBS and coverslipped using ProLong^®^ Diamond Antifade mounting media. After this first round of immunohistochemistry, sections were imaged on a Zeiss Z2 Axioimager (20×) using MetaSystems VSlide acquisition software and MetaCyte stitching software. Sections were then de-coverslipped for AD pathology staining as described above. Sections were imaged after this second round of immunohistochemical staining using the same imaging system.

Images from both staining rounds were opened on VSViewer v2.1.112 and individual channel images extracted from three areas of grey matter per section. Hoechst-stained nuclei in each labelling round were used as intrinsic markers for image registration. Hoechst images were first pre-processed (smoothed) in ImageJ (v1.52p) by applying a 50-pixel rolling background subtraction and a 5-pixel median filter. The processed nuclear images were then registered to each other using a custom-designed Python code. Jupyter Notebook was used to implement an AKAZE affine registration, and a transformation matrix was extracted and applied to all the individual images in the set [55]. These six channel images were used for the amyloid beta spatial analysis.

### Quantification using MetaMorph custom image analysis pipelines

Four novel custom image analysis journals were developed in MetaMorph software (Molecular Devices) to quantify Iba1-MOI populations with respect to AD pathology in normal and AD MTG. Journals for tissue-wide integrated intensity analysis, single-cell Iba1-MOI analysis, and amyloid beta spatial analysis are detailed in Additional file 1: Figure S1. All image analysis journals, and validation of their accuracy, are described in detail in the supplementary materials.

The *tissue*-*wide integrated intensity analysis* measured the integrated intensity of each MOI within all Iba1-MOI cells in the MTG. Binary masks of both the Iba1 staining and the MOI staining were generated using an adaptive threshold tool and combined to create a “master mask” containing all Iba1-MOI cells. MOI integrated intensity within the master mask was measured. MOI integrated intensity data were normalised to the region of interest tissue area and are thus equivalent to the concentration of the MOI in the tissue.

The *single cell Iba1*-*MOI analysis* measured the single-cell average intensity of Iba1 and the MOI from each Iba1-MOI co-label. The Iba1-MOI “master mask” described above was used. Each object within the master mask was considered a ‘cell’, and the average intensity of Iba1 and the MOI measured in each cell. Average intensity per Iba1-MOI cell is equivalent to the protein concentration per cell. Iba1 and MOI average intensities were plotted on an *XY* scatter plot. All Iba1-MOI cells from all AD and normal cases were pooled and Iba1-MOI populations segregated using a freehand tool, generating gates. Three Iba1-MOI populations were gated; Iba1^low^ MOI^high^, Iba1^high^ MOI^high^, and Iba1^high^ MOI^low^. The three population gates were transferred from the pooled Iba1-MOI plot to the *xy* scatter plot for each normal and AD case. The abundance of cells in each Iba1-MOI population (as a proportion of total Iba1-MOI cells per case) and the mean population intensity of each MOI were measured. Changes in the proportions of cells within each of the three Iba1-MOI populations reflected changes in Iba1 and MOI expression relative to one another. There was no difference in the overall number of Iba1-MOI cells between normal and AD cases (Additional file 1: Table S3). The mean intensity of each MOI population, equivalent to mean fluorescence intensities for flow cytometry data, was used to identify changes in MOI expression parallel to changes in Iba1 expression.

The *AD pathology load analysis* measured the load of amyloid beta and tau in the MTG. Binary masks of amyloid beta and tau staining were generated using the adaptive threshold tool, and the area of each mask was measured. Data are presented as the mean percentage area of amyloid beta or tau staining across the region of interest against the abundance of each Iba1^low^ MOI^high^ population.

The *amyloid beta spatial analysis* was a modified version of the single-cell Iba1-MOI analysis. Iba1 and MOI average intensity, and distance between each Iba1-MOI cell and its nearest amyloid beta plaque, was measured. Within each cell in the master mask, the intensity of a binary mask of amyloid beta binary was measured. If the intensity of the amyloid beta mask within a cell was above 0, the cell was on or interacting with an amyloid beta plaque and assigned as ‘plaque’. The amyloid beta master mask was subsequently dilated circularly in 5 µm increments. After each dilation, the intensity of the dilated amyloid beta mask was measured within each cell in the master mask. The dilations were discontinued at 50 µm. The distances were grouped as “plaque”, “plaque-adjacent” (up to 50 µm from a plaque) or “non-plaque” (greater than 50 µm from a plaque).

### Statistical analysis

F test of equality of variances and the Shapiro–Wilk normality were used to determine whether the variances between the two groups being compared were equal and if the data were normally distributed, respectively. If the variances were equal and the data were normally distributed, parametric tests were used. The student’s t test was used to compare between two groups, while the Pearson correlation was used for correlations of measured values with disease pathology, age, and post-mortem delay. The effect of sex was tested by one-way ANOVA with Holm–Sidak’s multiple comparisons. For non-parametric data, the Mann–Whitney test was used to compare between two groups, while the Spearman correlation was used for correlations of measured values with disease pathology, age, and post-mortem delay. The effect of sex was tested by the Kruskal–Wallis test with Dunn’s multiple comparisons. There were no significant correlations of any measured values with age, post-mortem delay, or sex in the normal or AD groups. Thus, these data were not discussed further. All data are presented as mean ± standard deviation, with statistical significance set at *p* ≤ 0.05. Correlations were considered strong if r ≥ 0.8 and moderate if 0.8 ≥ r ≥ 0.7.

## Results

### An Iba1^low^ myeloid cell population is more abundant in the AD temporal cortex

**Fig. 1:**
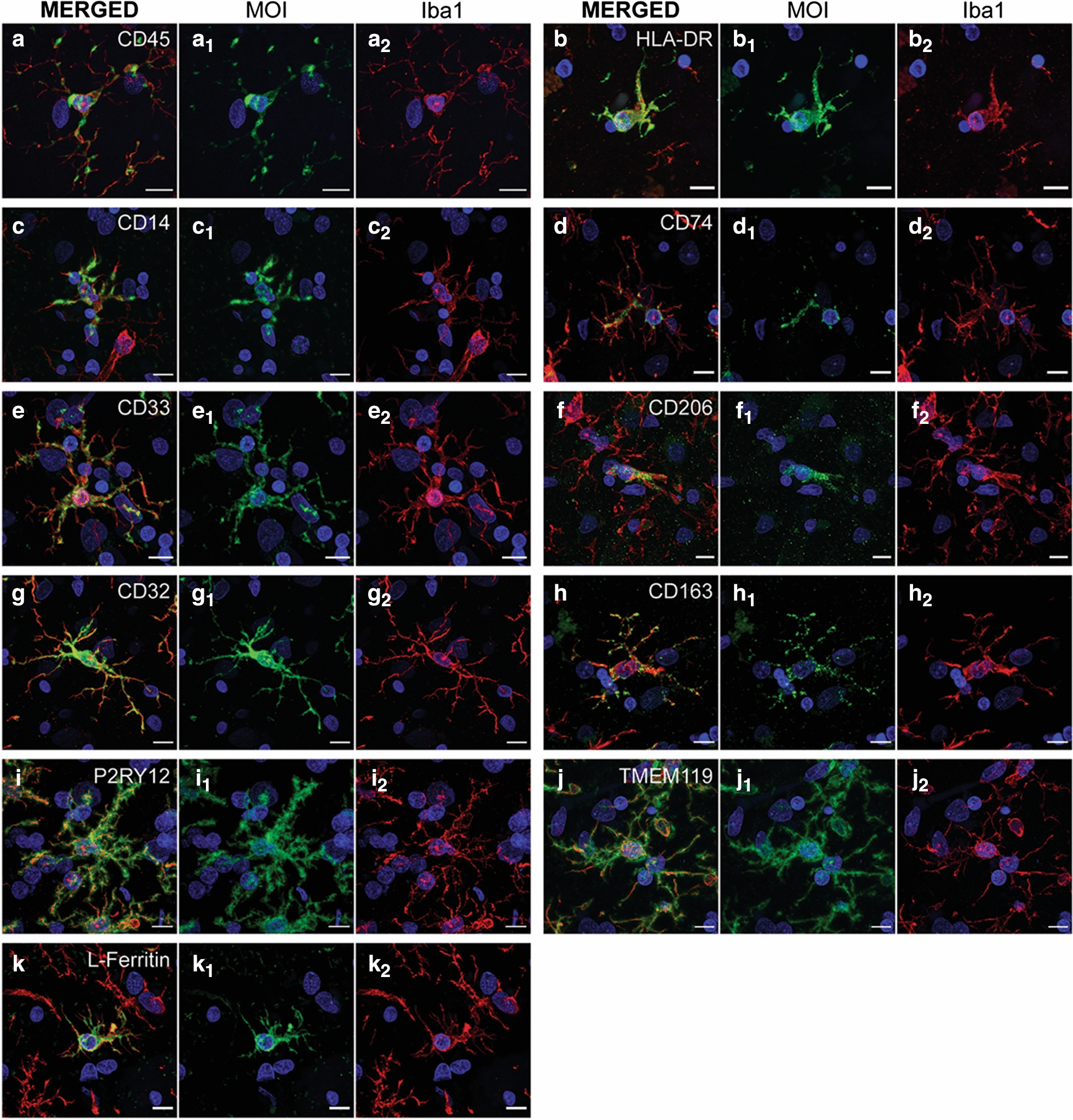
Co-labelling of MOIs with Iba1 in the human middle temporal gyrus. Pan myeloid cell marker, Iba1, was immunofluorescently co-labelled with one of the 11 MOIs investigated in the normal and AD human middle temporal gyrus; CD45 (**a**), HLA-DR (**b**), CD14 (**c**), CD74 (**d**), CD33 (**e**), CD206 (**f**), CD32 (**G**), CD163 (**h**), P2RY12 (**I**), TMEM119 (**j**), and L-Ferritin (**k**). Representative images are maximum projections of confocal *z*-stacks to demonstrate co-labelling of each MOI (green) co-labelling and Iba1 (red) with a Hoechst counterstain (blue); scale bars = 10 µm

We co-labelled Iba1 with one of 11 myeloid cell markers using immunohistochemistry on normal and AD MTG: CD45, HLA-DR, CD14, CD74, CD33, CD206, CD32, CD163, P2RY12, TMEM119, or L-Ferritin. All 11 myeloid cell markers were specifically expressed by Iba1-positive cells in both the normal and AD MTG (Fig. 1). Before performing more comprehensive analyses, we measured the total integrated intensity across the sections to determine changes in tissue-wide expression in AD (Additional file 1: Figure S2). Of the 11 MOIs, only the expression of CD45 was significantly increased in AD (Additional file 1: Figure S2).

**Fig. 2:**
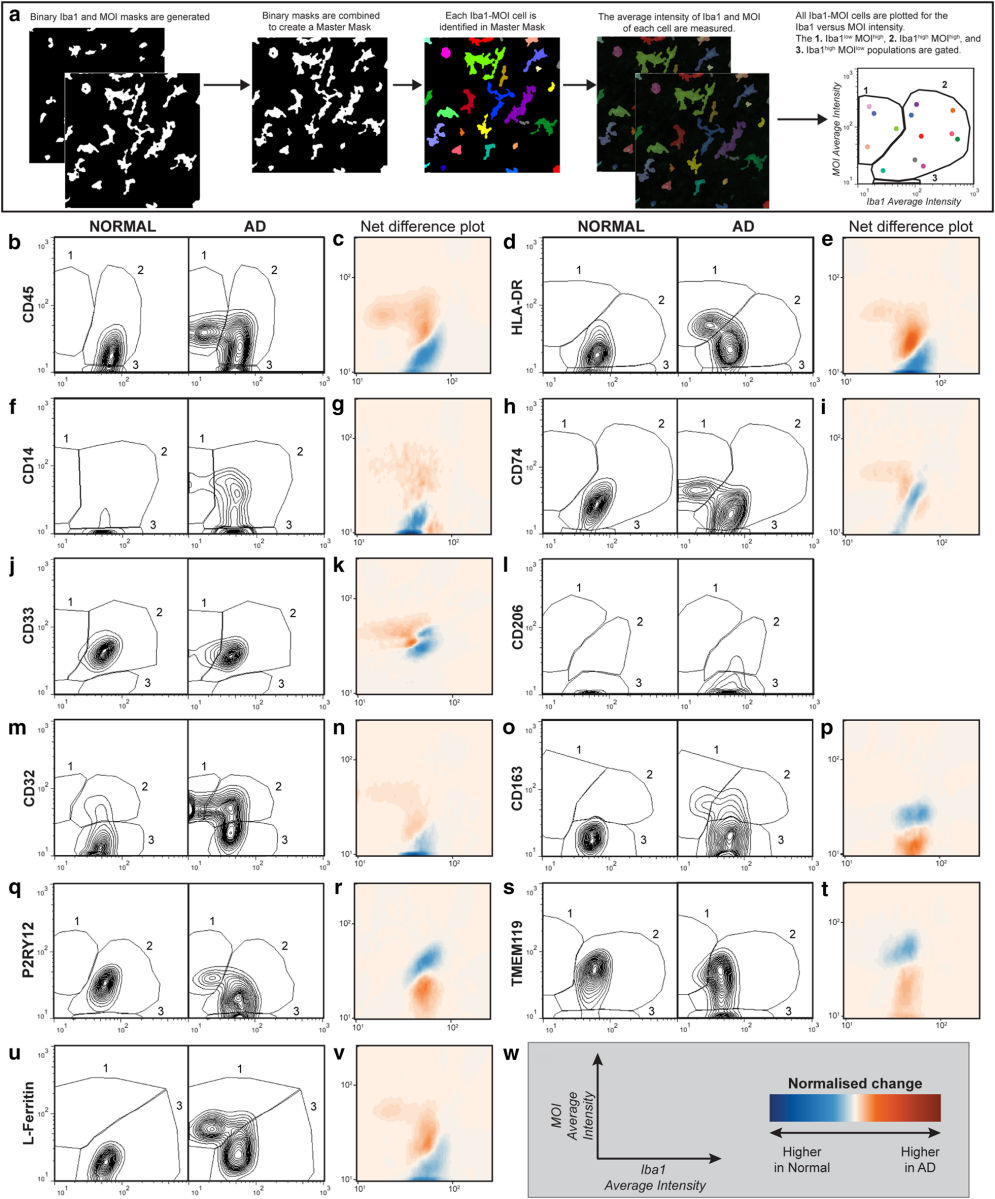
Novel single cell analysis identifies change in Iba1-MOI cell populations in AD. Iba1 was immunofluorescently co-labelled with markers in the normal and AD human middle temporal gyrus. The single cell Iba1-MOI analysis was used to quantify changes in the proportions of three Iba1-MOI populations in AD: 1. Iba1^low^ MOI^high^, 2. Iba1^high^ MOI^high^, and 3. Iba1^high^ MOI^low^ (**a**). The MOIs immunohistochemically stained for and quantified included CD45 (**b**, **c**), HLA-DR (**d**, **e**), CD14 (**f**, **g**), CD74 (**h**, **i**), CD33 (**j**, **k**), CD206 (**l**), CD32 (**m**, **n**), CD163 (**o**, **p**), P2RY12 (**q**, **r**), TMEM119 (**s**, **t**), and L-Ferritin (**u**, **v**). Example contour plots of Iba1 against MOI average intensity with the gates of the Iba1-MOI populations are presented for one normal and one AD case for CD45 (**b**), HLA-DR (**d**), CD14 (**f**), CD74 (**h**), CD33 (**j**), CD206 (**l**), CD32 (**n**), CD163 (**p**), P2RY12 (**q**), TMEM119 (**s**), and L-Ferritin (**u**). The net change in the distribution of the Iba1-MOI average intensities in AD is presented as a heat map of the normalised change (**c**, **e**, **g**, **i**, **k**, **m**, **o**, **r**, **t**, **v**). The *x*- and *y*-axis labels and heatmap scale are presented in (**w**)

Given that microglial sub-populations with unique transciptomic signatures have been identified in human AD brain tissue with single-cell RNA sequencing technologies [16–19], we sought to investigate microglial protein expression changes in immunohistochemically stained tissue at a single-cell level. We developed a custom MetaMorph image analysis pipeline which allowed for single-cell measurements of Iba1 and MOI staining intensity in post-mortem human tissue (Fig. 2A). The individual cell expression data was treated like FACS data (Fig. 2): for each Iba1-MOI co-label, we plotted all Iba1-MOI cells on an *XY* scatter plot based on their Iba1 and MOI average intensities and manually gated Iba1-MOI populations. For 10 of the 11 MOIs (CD45, HLA-DR, CD14, CD74, CD33, CD206, CD32, CD163, P2RY12, and TMEM119), three Iba1-MOI populations were identified: 1. Iba1^low^ MOI^high^, 2. Iba1^high^ MOI^high^, and 3. Iba1^high^ MOI^low^ (Fig. 2B, D, F, H, J, L, M, O, Q, and S). In contrast, for L-Ferritin only two Iba1-L-Ferritin populations were identified (labelled as 1 and 3): 1. Iba1^low^ L-Ferritin^high^ and 3. Iba1^high^ L-Ferritin^low^ (Fig. 2U). This finding indicates that the Iba1^low^ population was best delineated by high L-ferritin expression: if a cell expressed Iba1 at a low level it always expressed L-ferritin at a high level, and vice versa. Therefore, the Iba1^low^ population was not uniquely identified by high expression of one or a subset of the myeloid cell markers investigated.

**Fig. 3:**
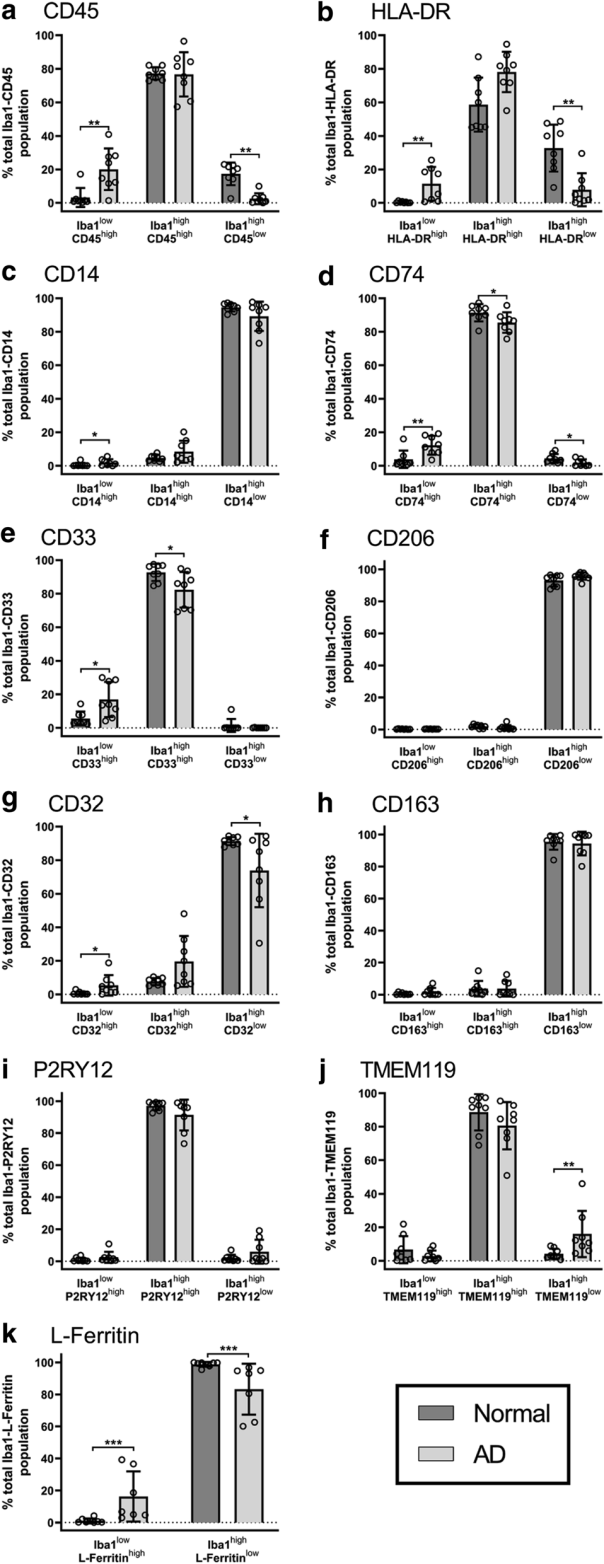
Iba1^low^ MOI^high^ populations are increased in AD. Iba1-MOI populations were identified by immunofluorescent co-labelling of Iba1 with markers CD45 (**a**), HLA-DR (**b**), CD14 (**c**), CD74 (**d**), CD33 (**e**), CD206 (**f**), CD32 (**g**), CD163 (**h**), P2RY12 (**I**), TMEM119 (**j**), and L-Ferritin (**k**). Following the single cell Iba1-MOI analysis, the abundance of the Iba1^low^ MOI^high^, Iba1^high^ MOI^high^, and Iba1^high^ MOI^low^ populations were quantified as a percentage of the total Iba1-MOI population identified in each normal and AD case. The abundance of each Iba1-MOI population was compared between control and AD using a Mann–Whitney test. Data are presented as mean abundance ± SD (n = 7 or 8). Significance of differences between normal and AD: ***p ≤ 0.001, **p ≤ 0.01, *p ≤ 0.05

For all myeloid cell markers, the intensity distributions of cells positive for Iba1 and/or the MOIs changed in AD, as reflected in the net difference plot for each marker (Fig. 2, Additional file 1: Table S4). When quantified, the most notable change in the abundances of the Iba1-MOI populations identified was a significant increase in the proportion of the Iba1^low^ MOI^high^ population for seven of the 11 MOIs in AD (Fig. 3): CD45, HLA-DR, CD14, CD74, CD33, CD32, and L-Ferritin. The increase in these seven Iba1^low^ MOI^high^ populations was typically accompanied by a reduction in the respective Iba1^high^ MOI^low^ populations, albeit this reduction did not reach significance for all seven of these MOIs (Fig. 3A–E, G, K). This suggests a model for the emergence of an Iba1^low^ population or populations in AD, at the expense of an Iba1^high^ population or populations. No significant changes in the Iba1-CD206, Iba1-CD163 and Iba1-P2RY12 populations were identified in AD (Fig. 3H, I). In addition, a significant increase in the Iba1^high^ TMEM119^low^ population was observed in AD, although this change was not accompanied by significant changes in the Iba1^high^ TMEM119^high^ or Iba1^low^ TMEM119^high^ populations (Fig. 3K).

### Iba1^low^ myeloid cells highly express activation markers

**Fig. 4:**
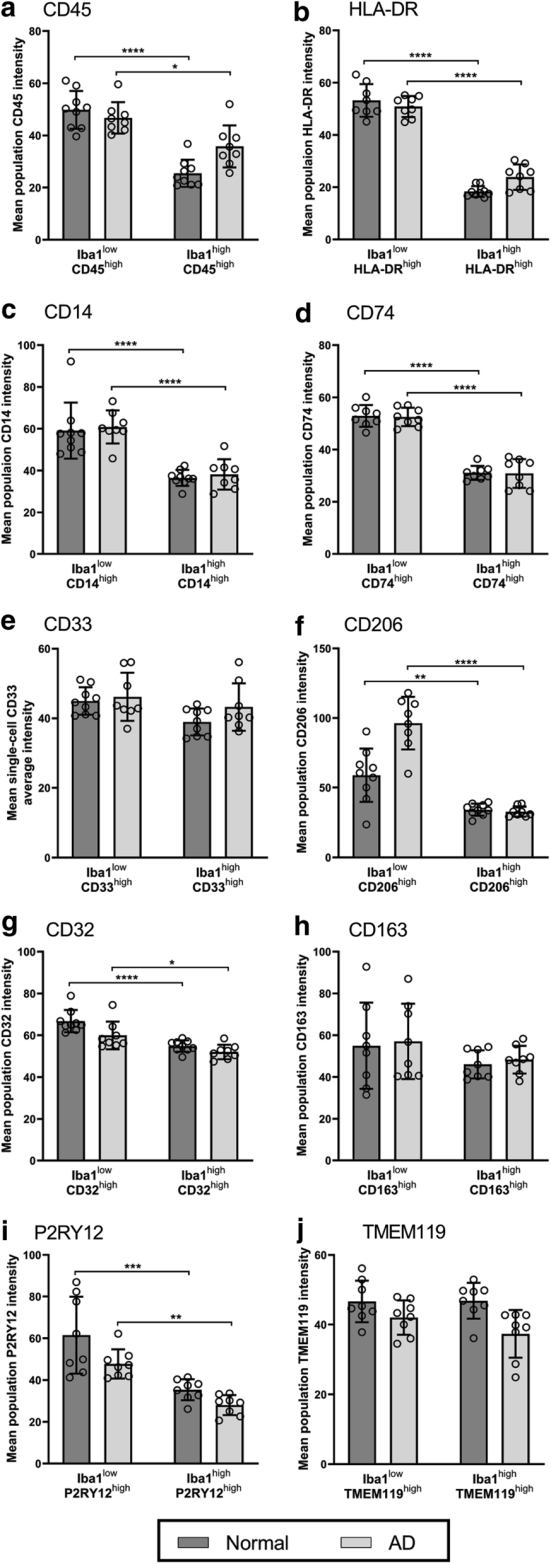
Iba1^low^ MOI^high^ population shows higher expression of activation markers than the respective Iba1^high^ MOI^high^ populations. Iba1-MOI populations were identified by immunofluorescent co-labelling of Iba1 with markers CD45 (**a**), HLA-DR (**b**), CD14 (**c**), CD74 (**d**), CD33 (**e**), CD206 (**f**), CD32 (**g**), CD163 (**h**), P2RY12 (**I**), and TMEM119 (**J**). The mean population intensity of the markers was measured for the Iba1^low^ MOI^high^ and Iba1^high^ MOI^high^ populations in each normal and AD case. The mean population intensities of each MOI were compared between the Iba1-MOI populations, and normal and AD using a two-way ANOVA with Tukey’s multiple comparisons test. Data are presented as mean ± SD (n = 7-8). Significance of differences between Iba1-MOI populations, and normal and AD: ****p ≤ 0.0001, ***p ≤ 0.001, **p ≤ 0.01, *p ≤ 0.05

Next, we sought to further characterise the Iba1^low^ MOI^high^ population, the most changed population in AD, to understand how they differed from their respective Iba1^high^ MOI^high^ population (Fig. 4). We calculated the mean population intensity for each MOI by determining where the centre of each Iba1-MOI population was on the contour plots presented in Fig. 2. We used the mean population intensities for each marker to determine differences in marker expression between the Iba1^low^ MOI^high^ and Iba1^high^ MOI^high^ populations in normal and AD MTG. It is important to note that the mean single-cell L-Ferritin average intensity was not compared between the Iba1-L-Ferritin populations as we did not identify an Iba1^high^ L-Ferritin^high^ population.

Of the seven MOIs with an increase in the Iba1^low^ population in AD, the mean population intensity of CD45, HLA-DR, CD14, CD74, and CD32 was significantly higher in the Iba1^low^ MOI^high^ population relative to the Iba1^high^ MOI^high^ population in both normal and AD, suggesting that cells with the highest MOI expression were those with low Iba1 (Fig. 4A–D, G). These MOIs are generally associated with microglial activation functions. While we identified no increase in the Iba1^low^ CD206^high^ population in AD, the mean population intensity of CD206 was higher in the Iba1^low^ CD206^high^ population relative to the Iba1^high^ CD206^high^ population in both normal and AD (Fig. 4F).

There was no increase in the abundance of the Iba1^low^ P2RY12^high^ and Iba1^low^ TMEM119^high^ populations in AD, distinguishing P2RY12 and TMEM119 from the other MOIs. However, unexpectedly, in both normal and AD cases the mean population intensity of P2RY12 was increased in the Iba1^low^ P2RY12^high^ population relative to the Iba1^high^ P2RY12^high^ population in both normal and AD cases (Fig. 4I). In this way, P2RY12 aligns with the MOIs generally associated with microglial activation functions. In contrast, there was no change in the mean single-cell TMEM119 average intensity between the Iba1^low^ TMEM119^high^ and Iba1^high^ TMEM119^high^ populations in normal and AD cases (Fig. 4J).

The changes in the proportions of Iba1-MOI populations demonstrate an increase in the expression of MOIs and a reduction in Iba1. The mean MOI average intensities per cell also demonstrate that the reduced Iba1 expression observed in AD is accompanied by an increase in the expression of a subset of MOIs. The subset of MOIs that is increased in this Iba1^low^ population were CD45, HLA-DR, CD14, CD74, CD206 and CD32, which have previously been associated with microglial activation (Additional file 1: Table S1).

### The abundance of the Iba1^low^ myeloid cell populations correlated with tau load but not amyloid beta

Next we correlated the abundance of the Iba1^low^ MOI^high^ populations with amyloid beta and tau load in AD cases (Table 2). Aside from CD74 and CD33, all the MOIs that showed an increase in the abundance of the Iba1^low^ MOI^high^ population in AD cases showed significant strong linear correlations with tau pathology load (Table 2). CD206, exclusively expressed by perivascular macrophages in the human brain, did not show an increase in the Iba1^low^ CD206^high^ population in AD. Despite this, the abundance of the Iba1^low^ CD206^high^ population significantly correlated strongly with tau pathology load (Table 2).

**Table 2:** Correlations for the abundance of the Iba1^low^ MOI^high^ populations and amyloid beta and tau load in AD cases

Marker	Amyloid beta		Tau	
	R value	*p* value	R value	*p* value
CD45	− 0.143	0.752	*0.810*	*0.022*
HLA-DR	0.0952	0.840	*0.905*	*0.005*
CD14	− 0.262	0.536	*0.833*	*0.015*
CD74	− 0.548	0.171	0.571	0.151
CD33	− 0.452	0.268	0.524	0.200
CD206	0.167	0.703	*0.833*	*0.015*
CD32	0.0476	0.935	*0.952*	*0.001*
CD163	0.238	0.582	0.619	0.115
P2RY12	− 0.214	0.619	0.667	0.096
TMEM119	− 0.0952	0.840	0.714	0.429
L-Ferritin	0.214	0.662	*0.896*	*0.003*

Italicised values were considered statistically significant (*p* ≤ 0.05)

**Fig. 5:**
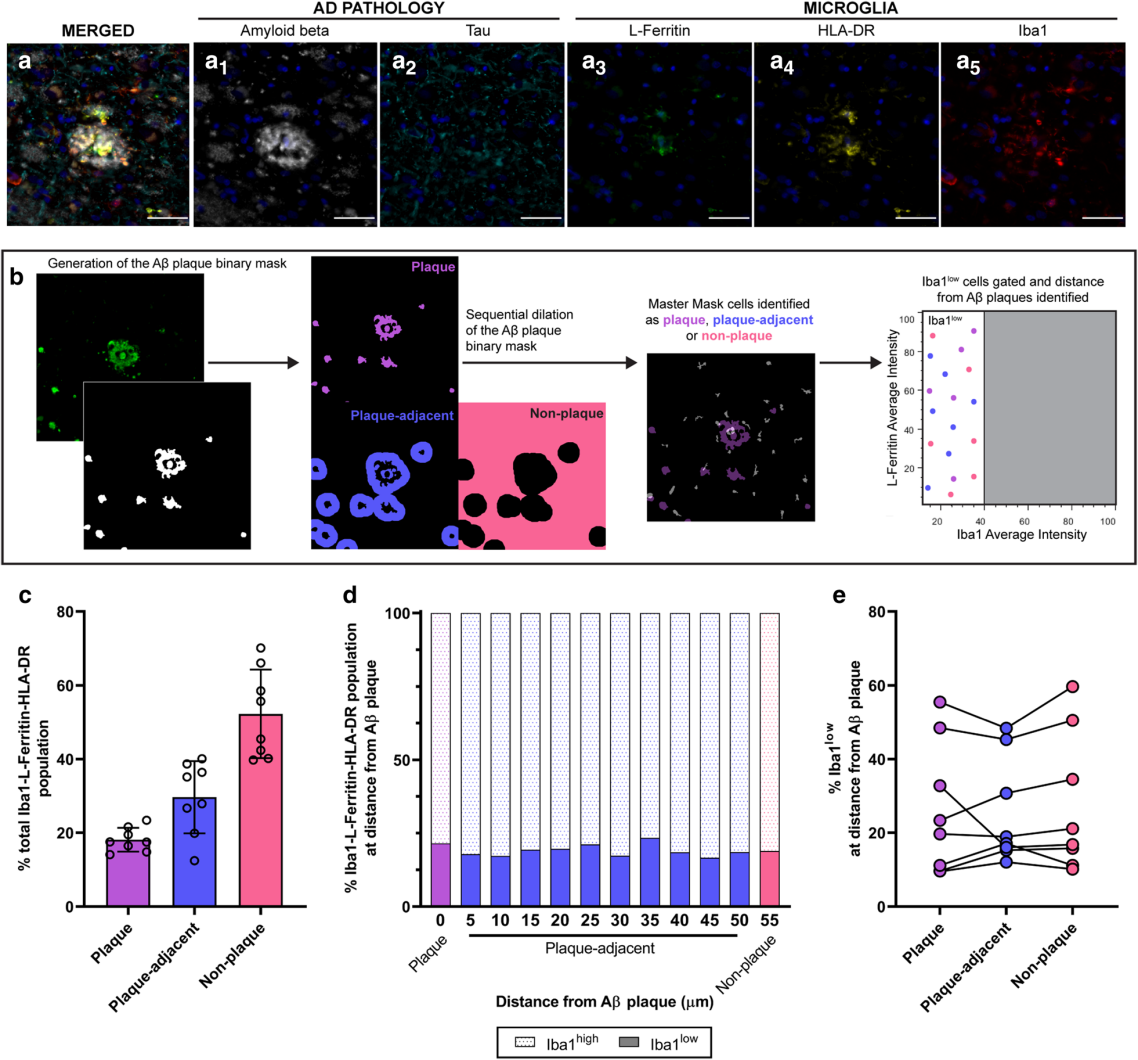
Iba1^low^ cells are not spatially distributed around Aβ plaques in AD cortex. To characterise the spatial distribution of Iba1^low^ cells relative to Aβ plaques, AD middle temporal gyrus sections were subjected to two rounds of immunofluorescent staining; (1) Iba1 was immunofluorescently co-labelled with L-Ferritin and HLA-DR, and (2) amyloid beta and tau. The images from the two rounds of staining were aligned, allowing us to determine the spatial localisation of Iba1-L-Ferritin-HLA-DR cells relative to amyloid beta plaques and tau tangles. Iba1-L-Ferritin-HLA-DR cells were identified near amyloid beta plaques but showed no spatial distribution around tau (**a**). A master mask of total Iba1-L-Ferritin-HLA-DR cells was generated. The location of Iba1-L-Ferritin-HLA-DR cells was classified as plaque, plaque-adjacent, or non-plaque (**b**). The proportion of total Iba1-L-Ferritin-HLA-DR cells at plaque, plaque-adjacent, and non-plaque spatial locations was quantified (**c**); data shown as mean ± SD (n = 8). The Iba1^low^ population was gated out from total Iba1-L-Ferritin-HLA-DR cells (as shown in **b**) and no difference in the abundance of Iba1^low^ cells at plaque, each 5 µm plaque-adjacent interval, and non-plaque spatial locations was identified (**d**); data shown as median percentage of total Iba1-L-Ferritin-HLA-DR cells at each spatial location (n = 8). When the 5–50 µm intervals were grouped into plaque-adjacent, the abundance of Iba1^low^ cells at plaque, plaque-adjacent, and non-plaque spatial locations remained unchanged (**e**); data shown as the percentage of total Iba1-L-Ferritin-HLA-DR cells at each spatial location with a single point per case (n = 8). The abundance of Iba1^low^ cells was compared between amyloid beta spatial locations using a repeated measures two-way ANOVA with the Geisser–Greenhouse correction and Tukey’s multiple comparisons test. *Aβ* amyloid beta

For all MOIs, the abundance of the Iba1^low^ MOI^high^ population did not correlate with amyloid beta load in AD (Table 2). We hypothesised that rather than correlating with overall amyloid beta load, the Iba1^low^ population might be spatially distributed around amyloid beta plaques. To test this hypothesis, we developed a spatial analysis pipeline to quantify the abundance of Iba1^low^ cells at different distances from amyloid beta plaques in AD MTG (Fig. 5A). To identify Iba1^low^ cells, Iba1 was co-labelled with two MOIs: L-Ferritin and HLA-DR. We chose these MOIs because high L-Ferritin expression best delineated the Iba1^low^ population, and HLA-DR is a MOI previously shown to be highly expressed by microglia around amyloid beta plaques [17, 49, 56]. Iba1-L-Ferritin-HLA-DR cells were identified as being plaque (on plaques), plaque-adjacent (5-50 µm from plaques), or non-plaque (> 50 µm from plaques).

We first quantified the proportion of total myeloid cells at each spatial location relative to amyloid beta plaques (Fig. 5B). Only 18.1 ± 3.22% of total Iba1-L-Ferritin-HLA-DR cells were located on plaques (Fig. 5C). We subsequently analysed the relative proportions of Iba1^low^ and Iba1^high^ cells at each spatial location relative to plaques (Fig. 5D, E), With no difference observed at different spatial locations (Fig. 5D, E). Therefore, the abundance of the Iba1^low^ population did not correlate with amyloid beta load and Iba1^low^ cells were not spatially distributed around amyloid beta plaques.

### Iba1^low^ microglia, but not perivascular macrophages, co-express L-Ferritin and activation MOIs

**Fig. 6:**
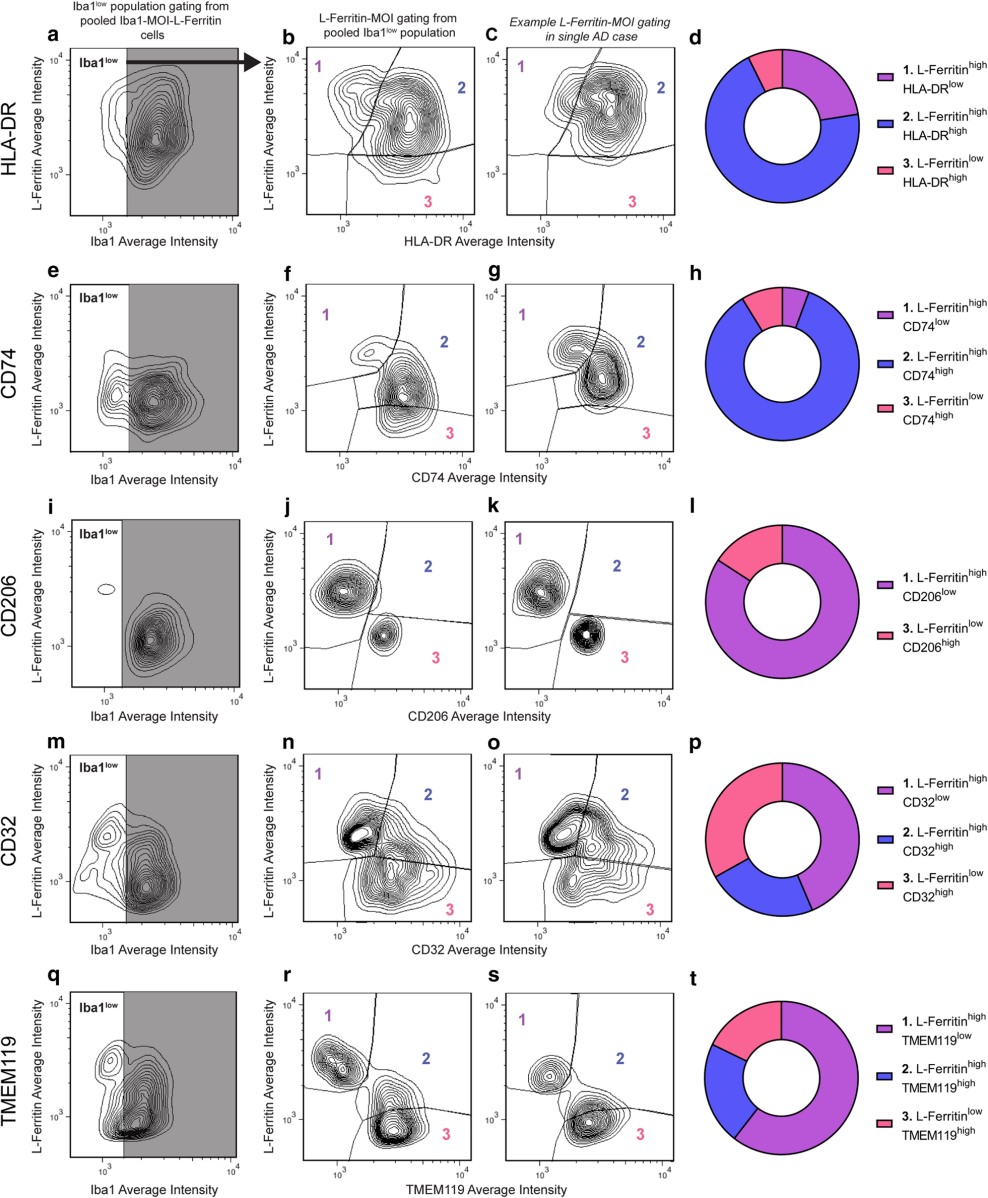
Iba1^low^ population co-expresses of L-Ferritin, HLA-DR, and CD74. To determine the co-expression of dysfunctional marker, L-Ferritin, with other immunophenotype MOIs on the Iba1^low^ population, Iba1 was immunofluorescently co-labelled with L-Ferritin and one other MOI; HLA-DR (**a**–**d**), CD74 (**e**–**h**), CD206 (**i**–**l**), CD32 (**m**–**p**), and TMEM119 (**q**–**t**). All Iba1-L-Ferritin-MOI cells were pooled from all normal and AD cases and plotted for their Iba1 average intensity against L-Ferritin average intensity to identify the Iba1^low^ population (**a**, **e**, **i**, **m**, **q**). All Iba1^low^ cells from all normal and AD cases were subsequently plotted for their MOI average intensity against L-Ferritin average intensity to identify three L-Ferritin-MOI populations; 1. L-Ferritin^high^ MOI^low^, 2. L-Ferritin^high^ MOI^high^, and 3. L-Ferritin^low^ MOI^high^ (**b**, **f**, **j**, **n**, **r**). An example of the L-Ferritin-MOI gating is shown on a single AD case for each MOI (**c**, **g**, **k**, **o**, **s**). The abundance of each L-Ferritin-MOI population was quantified as a percentage of total Iba1^low^ cells identified with each triple label (**d**, **h**, **l**, **p**, **t**); data presented as mean (n = 8)

While the Iba1^low^ population in AD was best delineated by high expression of L-Ferritin, a protein specifically expressed by dystrophic microglia, an Iba1^low^ CD206^high^ perivascular macrophage population was also identified [45, 57]. Thus, the Iba1^low^ population consists of both microglia and perivascular macrophages and is not fully identified by co-labelling of a single MOI with Iba1. We sought to determine the overlap of different Iba1^low^ MOI^high^ populations and their myeloid cell identity by triple-labelling Iba1, L-Ferritin, and one of a subset of MOIs: HLA-DR (Fig. 6A–D), CD74 (Fig. 6E–H), CD32 (Fig. 6I–L), CD206 (Fig. 6M–P), and TMEM119 (Fig. 6Q–T). This subset of MOIs was chosen because they span different functional states and have different expression patterns across microglia and perivascular macrophages (Additional file 1: Table S1). We used the Iba1-MOI single-cell image analysis pipeline (generating a master mask by combining the binary masks of Iba1, L-Ferritin and the MOI), gated out only the Iba1^low^ cells from the pooled Iba1-L-Ferritin-MOI population, and then gated the L-Ferritin-MOI populations within the Iba1^low^ cells. This triple-labelling and gating was carried out in both normal and AD to ensure accurate identification of the L-Ferritin^high^ MOI^low^, L-Ferritin^high^ MOI^high^, and L-Ferritin^low^ MOI^high^ populations, but the proportions of the three Iba1^low^ L-Ferritin-MOI populations were only quantified in AD cases.

Within the Iba1^low^ population, HLA-DR and CD74 showed high co-expression with L-Ferritin (Fig. 6A–H). Quantification of L-Ferritin and HLA-DR co-expression in the Iba1^low^ population demonstrated that 69.55 ± 29.47% of Iba1^low^ cells highly expressed both HLA-DR and L-Ferritin (Fig. 6D). The remaining single highly expressing population were primarily L-Ferritin^high^, constituting 22.26 ± 32.96% of total Iba1^low^ cells (Fig. 6D). Quantification of L-Ferritin and CD74 co-expression demonstrated that 84.97 ± 9.705% of Iba1^low^ cells highly expressed both CD74 and L-Ferritin (Fig. 6H). No co-expression of CD206 and L-Ferritin was observed on the Iba1^low^ population (L-Ferritin^high^ CD206^low^ = 82.89 ± 12.54% and L-Ferritin^low^ CD206^high^ = 15.59 ± 12.24%, Fig. 6I-L). This was expected with CD206 being a perivascular macrophage-specific marker in the human brain and no L-Ferritin expression on perivascular macrophages having been previously identified [57].

Iba1^low^ cells were somewhat evenly distributed in the L-Ferritin^low^ CD32^high^, L-Ferritin^high^ CD32^high^ and L-Ferritin^high^ CD32^low^ populations (Fig. 6M–P); a high percentage of Iba1^low^ cells were identified as either L-Ferritin^high^ CD32^low^ or L-Ferritin^low^ CD32^high^ with only a small percentage of Iba1^low^ cells highly expressing both L-Ferritin and CD32 (L-Ferritin^high^ CD32^low^ = 42.18 ± 17.52% and L-Ferritin^low^ CD32^high^ = 32.01 ± 13.60%, Fig. 6P; L-Ferritin^high^ CD32^high^ = 22.53 ± 12.57%, Fig. 6P). This may be attributed to the high CD32 expression by both perivascular macrophages and microglia in the AD brain [57]. Microglial-specific marker, TMEM119, also showed little to no co-expression with L-Ferritin, with only 21.41 ± 18.64% of Iba1^low^ cells highly expressing TMEM119 and L-Ferritin (Fig. 6Q–T). Therefore, while both TMEM119 and L-Ferritin are both specifically expressed by microglia, they are not expressed by the same Iba1^low^ microglial population.

## Discussion

In this study, we used novel quantification methods to determine the expression of 11 microglial proteins in post-mortem human AD cortex. Using a novel single-cell image analysis pipeline, we identified Iba1^low^ MOI^high^ microglial populations that were increased in the AD cortex. Further investigation of the Iba1^low^ MOI^high^ populations revealed that their abundances correlate with tau pathology load. Iba1^low^ myeloid cells were best delineated by high expression of L-Ferritin, and highly co-expressed L-Ferritin, CD74, and HLA-DR, a phenotype that reflects dysfunction. The results presented in this study are summarised in Additional file 1: Figure S3.

While the Iba1^low^ population was best delineated by high L-Ferritin expression, Iba1^low^ L-Ferritin^high^ cells also highly expressed both HLA-DR and CD74. This protein expression signature parallels the transcriptomic signature of the human AD microglial subpopulation identified using single-cell transcriptomic technologies [17]. The AD pathology-associated microglial population was one of four microglial subpopulations identified in normal and AD human prefrontal cortex using single nuclear RNA sequencing technology. This microglial subpopulation was overrepresented in high pathology load AD cases and was enriched with 77 transcripts relative to the other three microglial subpopulations, including MOIs L-Ferritin, HLA-DR, CD74, and CD14 that we investigated in this study [17]. Therefore, our results provide immunohistochemical validation of this AD pathology-associated population, although our spatial analysis shows these microglia are not specifically clustered near plaques.

In the human brain, L-Ferritin is a specific marker of dystrophic, dysfunctional microglia [10, 45, 57]. The L-Ferritin^high^ population identified both here and by Mathys et al. [17] is therefore likely to be dysfunctional. This high expression of L-Ferritin implicates iron dysregulation as a key driver of microglial dysfunction in AD [11, 59–61]. Interestingly, the reduced Iba1 expression in this population also suggests dysfunction. Iba1 is key to microglial membrane ruffling and phagocytosis, and a reduction in expression could suggest perturbed phagocytic capabilities [47]. Therefore, the Iba1^low^ L-Ferritin^high^ phenotype is a unique immunohistochemical signature to identify dysfunctional microglia in the human AD brain.

We identified strong correlations between the abundance of the Iba1^low^ L-Ferritin^high^ and Iba1^low^ HLA-DR^high^ (and other Iba1^low^ MOI^high^) populations with tau load, but not with amyloid beta load. The correlations of the Iba1^low^ populations with tau load is likely indicative of an interaction between microglial function and aggregate pathology, where microglial dysfunction and loss of microglial trophic support is predicted to drive the formation of tau pathology [10, 45]. This relationship has been demonstrated in post-mortem human AD tissue utilising the predictability of tau spread according to Braak staging to investigate the temporal relationship between microglial dysfunction and regional pathology [10]. L-Ferritin-positive dystrophic microglia were identified in Braak Stage I middle temporal gyrus devoid of tau pathology and were more abundant in entorhinal cortex with significant neurodegeneration and tau pathology [10]. Microglial dysfunction and the related loss of trophic support, as demonstrated by high L-Ferritin immunoreactivity and dystrophic morphology, are believed to precede the neurodegeneration and tau pathology in AD. Furthermore, the extent of microglial dystrophy further increases as neurodegeneration advances [12]. Together, with previous temporal relations between microglial dystrophy and tau previously realised, the correlations identified here between the Iba1^low^ populations and tau pathology further support the conclusion that the Iba1^low^ population is a dysfunctional microglial population in AD.

Given that microglia are known to interact and phagocytose amyloid beta in AD, we expected a correlation between the AD-associated Iba1^low^ population and amyloid load [62–68]. We investigated the spatial relationship between the dysfunctional Iba1^low^ population and amyloid beta plaques [16, 17]. However, the Iba1^low^ population identified by high L-Ferritin and/or high HLA-DR expression was not especially spatially distributed close to amyloid beta plaques. This is in direct contrast to disease-associated microglia in AD mice, including those expressing high L-Ferritin, identified around AD plaques [16, 69]. Human disease-associated microglia may indeed interact with amyloid beta at a certain time point or time points in disease, but these interactions may not persist in end-stage AD human tissue. Alternatively, this difference may reflect species differences. While AD pathology-associated microglia populations highly express phagocytosis-associated genes, high L-Ferritin expression suggests that iron dysregulation in conjunction with amyloid beta phagocytosis drive AD pathology-associated microglial signatures [11, 59–61]. In the human AD brain, iron is heavily concentrated at the centre of amyloid beta plaques and has been implicated in the aggregation of amyloid beta fibrils, demonstrating the likelihood of amyloid beta-iron interactions in AD [70, 71]. Therefore, the interplay between multiple pathogenic processes in AD likely drive the dysfunctional signature identified here and the associated changes in microglial function.

The characteristics of the Iba1^low^ population could be interpreted as evidence for the pro-inflammatory hypothesis as well as the dysfunctional hypothesis. While it could be concluded that the Iba1^low^ populations we identified provide support for the dysfunctional hypothesis of AD, these Iba1^low^ populations could also be considered chronically active and neurotoxic. Indeed, the Iba1^low^ populations were best delineated by high L-Ferritin expression, but the Iba1^low^ populations also expressed high levels of other MOIs associated with activation functions. The Iba1^low^ populations showed higher mean MOI average intensities per cell of CD45, HLA-DR, CD14, CD74, and CD32 relative to their respective Iba1^high^ population. Therefore, the increased presence of the Iba1^low^ population in the human AD brain could support the contrasting pro-inflammatory hypothesis. However, changes in microglial morphology and phenotype typically associated with chronic activation can also occur as part of cell senescence [13]. Aged microglia express inflammatory proteins not expressed by homeostatic microglia and accumulate lipid molecules. This is a signature that is enhanced in AD pathology-associated microglia [16, 17, 19, 72]. Because the MOIs up-regulated by the Iba1^low^ population span numerous and somewhat polarising functions, these populations are showing signs of exaggerated microglial ageing with impaired or dysfunctional intercellular communications [13]. The exacerbated activation state of the Iba1^low^ population we identified does not necessarily reflect a functional activation state, but instead a dysfunctional one. The characterisation of this dysfunctional microglial population demonstrates the complexity of microglial changes in AD.

By identifying Iba1^low^ populations that expressed L-Ferritin, specific for microglia, or CD206, specific for perivascular macrophages, we confirmed that both myeloid cell populations contribute to the Iba1^low^ population in AD. Identifying an Iba1^low^ CD206^high^ population indicates that PVMs comprise a substantial proportion of the Iba1^low^ population. While perivascular macrophages do not express dysfunctional MOI L-Ferritin, the perivascular macrophage Iba1^low^ population exhibits low Iba1 expression, hypothesised to be a sign of dysfunction. The higher expression, by the Iba1^low^ MOI^high^ than Iba1^high^ MOI^high^ populations, of MOIs associated with activation was also observed with MOIs highly expressed by perivascular macrophages: CD14, CD206, and CD32 [23, 57, 73]. Given that perivascular macrophages phagocytose amyloid beta aggregating around cerebral blood vessels, and the finding of blood–brain barrier breakdown in AD, the maintenance of normal perivascular macrophage function in late AD seems unlikely [74, 75]. Microglia and perivascular macrophages have key phenotypic differences in the normal brain, including their signatures of dysfunction. Overall, our data suggest that both microglia and perivascular macrophages are dysfunctional in AD.

This is one of the first immunohistochemical studies to validate single cell RNA sequencing AD-associated microglial population signatures. The ability to do this is inherently due to the novel single-cell image analysis method we developed to identify the expression of each MOI. Previous immunohistochemical studies have relied on tissue-wide measures, like the tissue-wide integrated intensity measures presented in this study, which as we demonstrated, can dilute subtle AD-associated cell-by-cell changes observed in heterogeneous populations like microglia [48]. The heterogeneity of microglia in the human brain has been identified with single-cell RNA sequencing, including the complexity of microglial changes in the human AD brain, albeit without extensive spatial context [16–19]. Single-cell measurements in immunohistochemically stained post-mortem human tissue have allowed for the identification of microglial heterogeneity and phenotypic changes in AD with some anatomical context [49, 57]. To provide anatomical context to the disease-associated microglial populations identified, integration of immunohistochemical and single-cell RNA sequencing datasets must be achieved. Although no spatial relationship between the dysfunctional Iba1^low^ microglia and AD pathology was identified here, it establishes context for how image analysis capabilities can be harnessed to generate and analyse single-cell data in immunohistochemically labelled tissue.

## Conclusions

Our novel single-cell image analysis pipeline allowed for the identification of an Iba1^low^ microglial population in immunohistochemically labelled human brain. This population was best delineated by high L-Ferritin expression, leading to the hypothesis that this is a dysfunctional microglial population. The phenotype of this population mirrors that of a human AD pathology-associated microglial subpopulation previously identified in a single cell RNA sequencing study. As such, our immunohistochemical data support the microglial dysfunction hypothesis of AD.

## Supplementary information

**Additional file 1.** Supplementary file containing supplementary methods, image analysis validation, and supplementary tables and figures.

## Data Availability

The datasets used and/or analysed during the current study available from the corresponding author on reasonable request.

## Abbreviations

AD: Alzheimer’s disease
MTG: Middle temporal gyrus
MOI: Marker of interest
CD: Cluster of differentiation
Iba1: Ionised calcium binding adaptor protein 1
HLA-DR: Human leukocyte antigen, DR isotype
Aβ: Amyloid beta
ROI: Region of interest
TSA: Tyramide signal amplification
