# Supplementary material for: Identification of a dysfunctional microglial population in human Alzheimer’s disease cortex using novel single-cell histology image analysis
Source: Acta Neuropathol Commun. 2020 Oct 20;8:170. doi: 10.1186/s40478-020-01047-9 (PMC7576851; doi:10.1186/s40478-020-01047-9)
Supplement: Supplementary file 1 — Additional file 1. Supplementary file containing supplementary methods, image analysis validation, and supplementary tables and figures. [file 40478_2020_1047_MOESM1_ESM.docx]

**Supplementary materials**

**Identification of a dysfunctional microglial population in human Alzheimer’s disease cortex using novel single-cell histology image analysis**

Molly E. V. Swanson^1,2,#^, Emma L. Scotter^2,3^, Leon C. D. Smyth^2,4,+^, Helen C. Murray^1,2^, Brigid Ryan^1,2^, Clinton Turner^5^, Richard L. M. Faull^1,2^, Mike Dragunow^2,4^, Maurice A. Curtis^1,2,6^

**Novel image analysis journals in MetaMorph**

Custom image analysis journals were developed in MetaMorph software (Molecular Devices) for the quantification of Iba1-MOI populations in normal and AD MTG. Four journals were developed for this analysis: 1) integrated intensity analysis journal, 2) single-cell Iba1-MOI analysis journal, 3) AD pathology load journal, and 3) beta amyloid spatial analysis journal. The steps for these journals (apart from the AD pathology load journal) and subsequent analysis are summarized in Supplementary figure 1.

***Tissue-wide integrated intensity analysis journal***

Images from MOI-Iba1 co-labelling on free-floating MTG sections were used for the tissue-wide integrated intensity analyses. A region of interest (ROI) was drawn around all layers of the grey matter on the Hoechst image. By creating this ROI using the Hoechst counterstain, folds and tissue defects were excluded and no bias towards microglial staining occurred. The area outside this ROI was then cleared for Iba1 and marker MOI images. Next, Iba1- or MOI-positive microglia were identified based on size and staining intensity using the adaptive threshold processing tool. This allowed the creation of two binary microglial ‘masks’ and these binary masks were combined, creating a ‘master mask’. This master mask therefore highlighted all Iba1-positive and/or MOI-positive microglia within the ROI. Without the generation of the master mask to capture all Iba1 and/or MOI highly expressing cells, some cells expressing Iba1 and/or the MOI lowly would not have been analysed. Finally, the Iba1 and MOI staining within the master mask area was thresholded and the areas and integrated intensities were measured. Data are presented as integrated intensity normalized to ROI area, which is equivalent to the total concentration of Iba1 or MOI.

***Single-cell Iba1-MOI analysis journal***

Microglia are highly heterogeneous in tissue and as such it was hypothesized that tissue-wide integrated intensity measurements may not identify subtle disease-associated cell-to-cell changes. Therefore, a custom MetaMorph journal was developed, capable of measuring Iba1 and MOI staining intensity within each individual microglial cell in post-mortem human tissue.

Images from MOI-Iba1 co-labelling on free-floating MTG sections were used for the single-cell Iba1-MOI analyses. The steps within the Iba1-MOI population cell gating analysis journal were equivalent to those in the integrated intensity analysis up to the development of a master mask. However, more constrained size and staining intensity limits were used for the identification of microglia. This excluded some of the finer microglial processes from the analysis for a more accurate measure of total microglia number within the ROI. To identify each individual microglia as a single object, the master mask was used as a template and regions were drawn around each object or ‘cell’. Within each cell, the average intensity of both Iba1 and the MOI was measured on the respective images. In the same way that the integrated intensity across the tissue section normalised to ROI area can be considered equivalent to the total concentration of Iba1 or MOI, the average intensity per object (or the total amount of signal divided by the cell size) can be considered equivalent to the concentration of the protein within the cell. CSV files with single cell average intensity data were converted to FCS files on R (v 3.6.0.1).

Gating of Iba1-MOI populations was carried out in FlowJo (v 7.6.5). All cells identified in normal and AD cases were pooled and plotted in an *xy* scatter plot based on their Iba1 and MOI average intensities. Iba1-MOI populations were delineated with the freehand tool, generating gates. These gates were then applied to the cell populations from each case. By using the pooled populations from all normal and AD cases for the gates and subsequently applying these gates to each case, any AD-specific change gating bias was removed. Three Iba1-MOI populations were identified: 1. Iba1^low^ MOI^high^, 2. Iba1^high^ MOI^high^, and 3. Iba1^high^ MOI^low^. The proportion and the mean MOI population intensity within each Iba1-MOI population were measured. These measures reflected changes in Iba1 and MOI expression within the Iba1-MOI populations.

***AD pathology load journal***

Images from amyloid beta and tau pathology staining on free-floating MTG sections were used for the AD pathology load analysis. As with the Iba1-MOI quantification journals, an ROI was drawn around all layers of the grey matter on the Hoechst image where pathology was to be quantified. Within the ROI, all Aβ and tau was identified on their respective images using the adaptive threshold processing tool. This generated binary masks of Aβ and tau present within the ROI. The areas of the binary masks (as percentages of the total ROI) were used as measures of Aβ and tau load within each section.

***Amyloid beta spatial analysis journal***

The aligned images of Iba1, HLA-DR, L-Ferritin, amyloid beta, tau, and Hoechst from the paraffin immunohistochemistry were used for the amyloid beta spatial analysis. It is important to note that because of the homogeneous spread of tau pathology throughout the MTG, the spatial analysis was only carried out on amyloid beta. An ROI was drawn around all layers of the grey matter on the Hoechst image where microglia and pathology were to be quantified. The area outside the ROI was cleared on the Iba1, HLA-DR, L-Ferritin, and amyloid beta images. As with the single-cell Iba1-MOI analysis, a master mask was generated from Iba1, HLA-DR, and L-Ferritin binary masks, and each object within the master mask was considered a cell. Within each cell, the average intensity of Iba1, HLA-DR, and L-Ferritin was measured. To determine the spatial location of each of these cells relative to amyloid beta plaques, a binary mask of amyloid beta plaques was generated using the adaptive threshold tool. Within each cell in the master mask, the intensity of the amyloid beta binary mask was measured. If the intensity of the amyloid beta mask within a cell was greater than 0, the cell was on or interacting with an amyloid beta plaque and assigned as ‘plaque’. The amyloid beta master mask was subsequently dilated circularly in 5 µm increments, between which the intensity of the dilated amyloid beta mask was measured within each cell in the master mask. The dilations were discontinued at 50 µm. Each Iba1-HLA-DR-L-Ferritin cell in the master mask was assigned as plaque, plaque-adjacent (5-50 µm), or non-plaque (55 µm). These distances relative to amyloid beta plaques were investigated as they were equivalent to those investigated by Serrano-Ponzo et al. (2013), and allowed us to determine whether a spatial association of microglia with amyloid beta plaques could drive their phenotype.

All Iba1-HLA-DR-L-Ferritin cells were plotted in FlowJo based on their Iba1 and L-Ferritin average intensities, and all Iba1^low^ cells were manually gated. We analysed the proportion of Iba1^low^ to Iba1^high^ cells at each spatial location relative to amyloid beta plaques.

**Validation of custom MetaMorph image analysis journals**

Validation of MetaMorph journals was undertaken using manual counting in ImageJ. These manual counts were used to 1) validate the number of objects detected by the journals and to 2) confirm that the objects being counted by the journals were cells.

***Validating cell counting by custom journal***

Three random 1500-by-1500-pixel images from the CD74-Iba1 co-labelling from each case were used for validation. On an Iba1-CD74-Hoechst image, all Iba1-positive or CD74-positive cells were counted using the multipoint region tool. Cells were counted if they were positive for either Iba1 or CD74 and contained a Hoechst-positive nucleus. The point intensities of Iba1 and the MOI were subsequently measured. A cell was considered positive for Iba1 if it was 45 grey values higher than background and positive for CD74 if it was 40 grey values above, to give a final number of manually counted Iba1-CD74 cells. The number of objects in these 1500-by-1500-pixel images was also measured using the single-cell Iba1-CD74 analysis journal.

The number of Iba1-CD74 objects identified with the master mask in the MetaMorph journal significantly correlated with the number of Iba1-CD74 microglia counted manually (r = 0.8226, p < 0.0001, Supplementary Figure 4). Therefore, the custom journals detect an equivalent number of Iba1-CD74 cells to manual counting.

Next, the master mask’s ability to identify more microglia than either single marker binary mask alone was examined. More microglia per image were manually counted based on both Iba1 and MOI immunoreactivity versus only one marker (Iba1 only = 73.57 ± 31.91, CD74 only = 64.73 ± 25.22, Iba1-CD74 = 90.36 ± 34.07, p < 0.0001, Supplementary Figure 4). This was reflected in the automated analyses where more microglia were identified using the master mask in the MetaMorph journals versus Iba1-only or CD74-only masks (Iba1 only = 88.61 ± 30.19, CD74 only = 95.58 ± 35.92, master mask = 109.1 ± 30.73, p < 0.0001, Supplementary Figure 4). Therefore, the master mask in the automated analysis method is identifying a greater proportion of total microglia than single marker binary masks and this reflects what was observed with traditional manual counting methods.

***Validating cell discrimination by custom journal***

To determine whether the objects identified by the MetaMorph journal were the same as those manually counted, the multipoint regions in ImageJ were transferred on to the binary master masks. The intensities were measured on each master mask, where a value of either 0 (negative) or 65535 (positive) was measured. Of the cells manually counted and considered true microglia, 93.0 ± 5.78 % were also positively identified by the journal. This high sensitivity alongside the strong correlation between manually counted cells and the number of objects detected by the MetaMorph journal lead to the conclusion that these journals were identifying and measuring the Iba1-MOI microglia accurately.

| **Supplementary Table 1: Markers of interest investigated in this study with changes previously identified in Alzheimer’s disease.** | | |
| --- | --- | --- |
| **Marker of interest** | **Function** | **AD-associated changes** |
| **Iba1** | Calcium binding protein for membrane ruffling and phagocytosis; up-regulated during activation [40,46,47] | 50% of studies on post-mortem human brain show a significant increase in Iba1 in AD in at least one brain region [48]; Iba1^low^ HLA-DR^high^ population is significantly increased in AD brain [49]. |
| **CD45** | Modulation of inflammatory signals [50] | 67% of studies on post-mortem human brain show a significant increase in CD45 in AD in at least one brain region [48]; increased number of CD45-positive cells in the hippocampus and frontal cortex [20,51]; increased CD45 mRNA in the temporal cortex [21]. |
| **HLA-DR** | Antigen presentation [22]. | 84% of studies on post-mortem human brain show a significant increase in HLA-DR in AD in at least one brain region [48]; enriched in disease-associated microglia in mouse AD model [16]; enriched in human AD pathology-associated population [17]. |
| **CD14** | LPS co-receptor and phagocytosis [23,24]. | Increased CD14-positive cells surround amyloid beta plaques [23]; increased CD14 mRNA [25]; enriched in human AD pathology-associated population [17]. |
| **CD74** | Supports HLA-DR formation and transportation to the cell surface [26,27] | Increased CD74-positive cells in hyperphosphorylated tangles in AD hippocampus [28]; enriched in disease-associated microglia in mouse AD model [16]; enriched in human AD pathology-associated population [17]. |
| **CD33** | Mediate cell-to-cell interactions that modulate immune responses [6,29] | Increased number of CD33-positive cells in the AD frontal cortex [30]; increased CD33 mRNA in the AD frontal and temporal cortices and hippocampus [30–32]. |
| **CD206** | Phagocytosis and endocytosis of mannosylated enzymes and proteins[33] | Increased CD206 mRNA in the frontal lobe in AD [34]. |
| **CD32** | Binds to Fc regions of antibodies to mediate phagocytosis [35] | Increased number of CD32-positive cells associated with amyloid beta plaques in AD [36] |
| **CD163** | Clears extracellular haemoglobin haptoglobin complexes; binds and sequesters TNFα-like weak inducer of apoptosis [37]. | Increased CD163-positive cell density in the AD frontal and temporal cortices, and hippocampus [38,39]. |
| **P2RY12** | Detection of nucleotides [41]; microglial-specific expression in the brain [42]. | Reduced P2RY12 staining area in AD hippocampus in late-stage pathology [21]; reduced expression in disease-associated microglial population [16]. |
| **TMEM119** | Unknown; microglial-specific expression in the brain [43]. | Unchanged immunohistochemical staining area in AD frontal cortex [44]; reduced expression in disease-associated microglial population [16]. |
| **L-Ferritin** | Sequesters free iron to reduce the formation of reactive oxygen species [45]. | Increased L-Ferritin immunoreactivity precedes formation of tau tangles in AD [10]; enriched in human AD pathology-associated microglial population [17]. |

| **Supplementary Table 2: Primary antibodies, concentrations and visualisation methods** | | | |
| --- | --- | --- | --- |
| **Primary antibody** | **Dilution** | **Catalogue number, Company** | **Visualisation** |
| goat anti-Iba1 | 1:1,000 | ab5076, Abcam | Alexa Fluor® 594 or 647 secondary |
| rabbit anti-P2RY12 | 1:2,000 | HPA014518, Sigma-Aldrich | Alexa Fluor® 488 secondary |
| rabbit anti-TMEM119 | 1:500 | ab185333, Abcam | TSA Alexa Fluor® 488 |
| mouse anti-CD45 | 1:500 | ab8216, Abcam | TSA Alexa Fluor® 488 |
| mouse anti-HLA-DR | 1:500 | M0775, DAKO | Alexa Fluor® 488 or 594 secondary |
| rabbit anti-CD14 | 1:500 | ab133335, Abcam | TSA Alexa Fluor® 488 |
| rabbit anti-CD74 | 1:500 | ab64772, Abcam | Alexa Fluor® 488 or 594 secondary |
| mouse anti-CD33 | 1:100 | NCL-L-CD33, Novacastra | TSA Alexa Fluor® 488 |
| rabbit anti-CD206 | 1:1,000 | ab64693, Abcam | Alexa Fluor® 488 or 594 secondary |
| rabbit anti-CD32 | 1:500 | ab155972, Abcam | TSA Alexa Fluor® 488 |
| rabbit anti-CD163 | 1:1,000 | ab182422, Abcam | TSA Alexa Fluor® 488 |
| rabbit anti-L-Ferritin | 1:2,000 | F5012, Sigma-Aldrich | Alexa Fluor® 488 or 594 secondary |
| mouse anti-L-Ferritin | 1:2,000 | ab201945, Abcam | Alexa Fluor® 488 or 594 secondary |
| mouse anti-Amyloid beta | 1:100 | M0872, DAKO | Alexa Fluor® 488 |
| rabbit anti-Tau | 1:5,000 | A0024, DAKO | AlexaFluor® 594 |
| Rabbit anti-P-Ser Tau | 1:200 | Sc-101815, Santa Cruz | AlexaFluor® 594 |

**
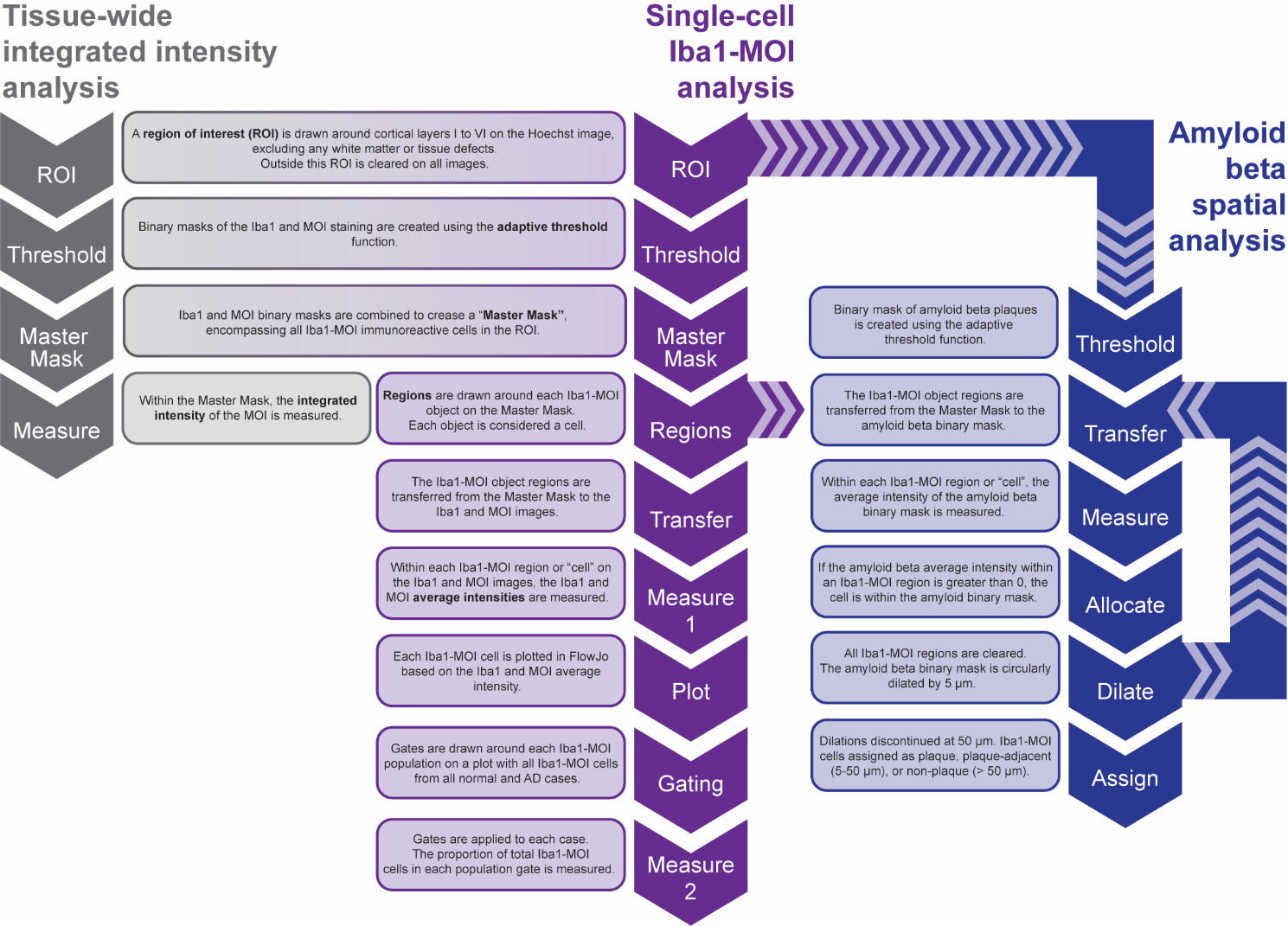
**

**Supplementary Figure 1: Flow diagram summarising the custom MetaMorph image analysis pipelines.**

The MetaMorph journal steps are shown for the tissue-wide integrated intensity analysis, the single-cell Iba1-MOI analysis, and the amyloid beta spatial analysis. Note that the amyloid beta spatial analysis is a modification of the single-cell Iba1-MOI analysis to measure the distance of each Iba1-MOI cell from amyloid beta plaques.

| **Supplementary Table 3: MOI-Iba1 cell densities in normal and AD MTG** | | | | |
| --- | --- | --- | --- | --- |
| **MOI** | **Mean MOI-Iba1 cells/mm^2^ (± SD)** | | **Statistical significance** | |
|  | **Normal** | **AD** | **P value** | **Summary** |
| CD45 | 236.6 ± 47.70 | 253.5 ± 44.79 | 0.2345 | ns |
| HLA-DR | 245.5 ± 80.71 | 200.1 ± 46.67 | 0.3823 | ns |
| CD14 | 199.7 ± 62.87 | 171.2 ± 43.18 | 0.4418 | ns |
| CD74 | 272.3 ± 46.43 | 255.7 ± 60.94 | 0.8785 | ns |
| CD33 | 222.6 ± 45.42 | 205.1 ± 46.86 | 0.3282 | ns |
| CD206 | 221.2 ± 51.54 | 185.7 ± 35.39 | 0.1049 | ns |
| CD32 | 180.9 ± 53.07 | 167.7 ± 34.01 | 0.5641 | ns |
| CD163 | 234.9 ± 53.21 | 203.3 ± 49.02 | 0.6454 | ns |
| P2RY12 | 241.1 ± 48.98 | 216.0 ± 40.63 | 0.2830 | ns |
| TMEM119 | 244.2 ± 42.66 | 199.0 ± 53.30 | 0.0821 | ns |
| L-Ferritin | 229.5 ± 41.46 | 225.6 ± 46.10 | 0.6943 | ns |

**
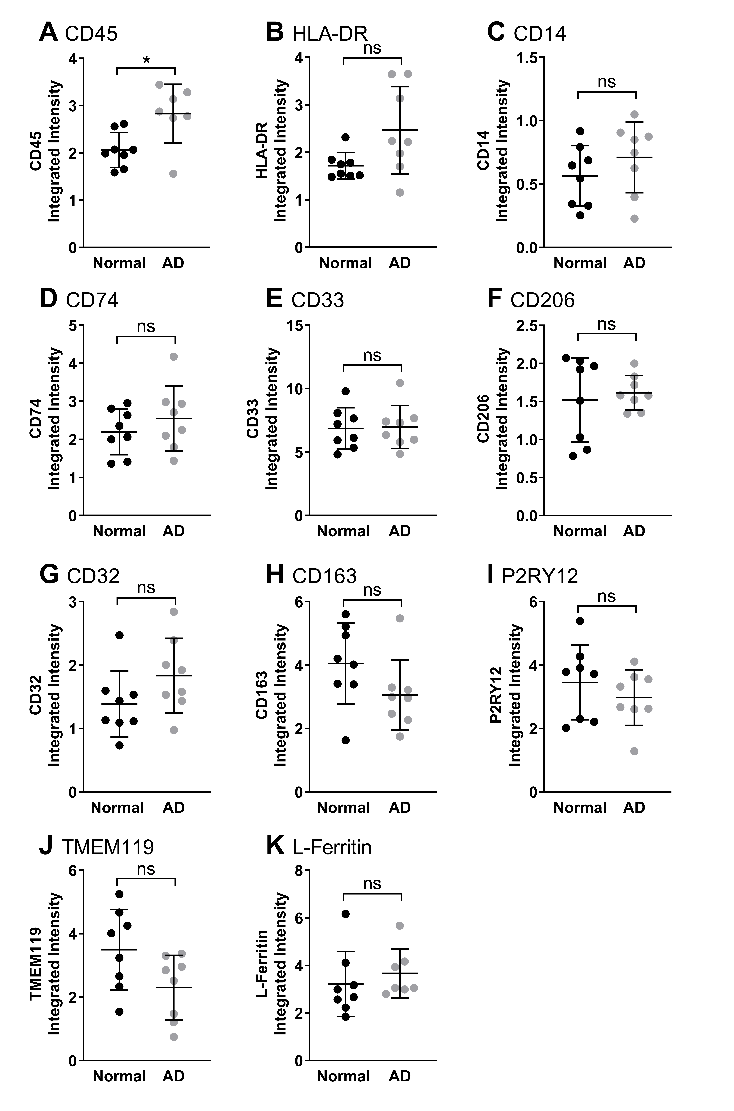
**

**Supplementary Figure 2: Tissue-wide quantification of MOI integrated intensities identifies no change in immunophenotype MOI abundance in normal and Alzheimer’s disease human middle temporal gyrus.**

Pan myeloid cell marker, Iba1, was immunofluorescently co-labelled with immunophenotype MOIs in the normal and AD human middle temporal gyrus. The integrated intensity analysis was used to quantify the expression of each MOI across the Iba1-MOI population. The MOIs immunohistochemically stained for and quantified were CD45 (A), HLA-DR (B), CD14 (C), CD74 (D), CD33 (E), CD206 (F), CD32 (G), CD163 (H), P2RY12 (I), TMEM119 (J), and L-Ferritin (K). The integrated intensity of each MOI was compared between normal and AD with a student’s t-test or a Mann-Whitney test; data are presented as mean integrated intensity ± SD (n = 8). Significance of differences between normal and AD: ***** p ≤ 0.05, **ns** not significant.

**Supplementary Table 4: Percentage of Iba1^low^ MOI^high^, Iba1^high^ MOI^high^, and Iba1^high^ MOI^low^ populations of total Iba1-MOI cells in normal and AD MTG.**

| MOI | Population | Percentage of total Iba1-MOI population | |  | Statistical significance | |
| --- | --- | --- | --- | --- | --- | --- |
|  |  | **Normal** | **AD** |  | **P value** | **P value summary** |
| P2RY12 | Iba1^low^ P2RY12^high^ | 0.9455 ± 1.31 | 2.429 ± 3.457 |  | 0.2753 | ns |
|  | Iba1^high^ P2RY12^high^ | 97.06 ± 2.728 | 91.35 ± 9.644 |  | 0.3416 | ns |
|  | Iba1^high^ P2RY12^low^ | 1.871 ± 2.21 | 5.989 ± 7.487 |  | 0.5737 | ns |
| TMEM119 | Iba1^low^ TMEM119^high^ | 6.662 ± 8.056 | 2.972 ± 3.204 |  | 0.5054 | ns |
|  | Iba1^high^ TMEM119^high^ | 88.64 ± 10.93 | 80.58 ± 14.11 |  | 0.1605 | ns |
|  | Iba1^high^ TMEM119^low^ | 4.187 ± 3.031 | 16.02 ± 13.77 |  | 0.0047 | ** |
| CD45 | Iba1^low^ CD45^high^ | 3.178 ± 5.653 | 20.07 ± 12.43 |  | 0.0070 | ** |
|  | Iba1^high^ CD45^high^ | 77.26 ± 3.705 | 76.69 ± 13.2 |  | 0.9364 | ns |
|  | Iba1^high^ CD45^low^ | 17.35 ± 6.783 | 2.4579 ± 3.248 |  | 0.0011 | ** |
| HLA-DR | Iba1^low^ HLA-DR^high^ | 0.393 ± 0.4596 | 11.17 ± 10.12 |  | 0.0011 | ** |
|  | Iba1^high^ HLA-DR^high^ | 58.68 ± 16.1 | 78.13 ± 12.04 |  | 0.0270 | * |
|  | Iba1^high^ HLA-DR^low^ | 32.71 ± 14.02 | 7.828 ± 9.865 |  | 0.0030 | ** |
| CD14 | Iba1^low^ CD14^high^ | 0.5753 ± 1.216 | 1.942 ± 1.962 |  | 0.0499 | * |
|  | Iba1^high^ CD14^high^ | 4.715 ± 1.773 | 8.451 ± 6.565 |  | 0.3823 | ns |
|  | Iba1^high^ CD14^low^ | 94.5 ± 2.499 | 89.23 ± 8.723 |  | 0.3416 | ns |
| CD74 | Iba1^low^ CD74^high^ | 3.881 ± 5.154 | 12.23 ± 5.621 |  | 0.0047 | ** |
|  | Iba1^high^ CD74^high^ | 91.36 ± 5.216 | 85.45 ± 6.097 |  | 0.0499 | * |
|  | Iba1^high^ CD74^low^ | 4.413 ± 2.614 | 1.832 ± 2.02 |  | 0.0499 | * |
| CD33 | Iba1^low^ CD33^high^ | 5.541 ± 4.173 | 16.91 ± 10.46 |  | 0.0148 | * |
|  | Iba1^high^ CD33^high^ | 92.65 ± 5.076 | 82.34 ± 10.6 |  | 0.0499 | * |
|  | Iba1^high^ CD33^low^ | 1.417 ± 3.873 | 0.05488 ± 0.1251 |  | 0.3579 | ns |
| CD206 | Iba1^low^ CD206^high^ | 0.199 ± 0.1627 | 0.2263 ± 0.0777 |  | 0.3823 | ns |
|  | Iba1^high^ CD206^high^ | 1.844 ± 1.06 | 1.119 ± 1.604 |  | 0.0830 | ns |
|  | Iba1^high^ CD206^low^ | 92.96 ± 3.642 | 95.8 ± 2.276 |  | 0.0876 | ns |
| CD32 | Iba1^low^ CD32^high^ | 0.737 ± 0.978 | 5.421 ± 6.084 |  | 0.0379 | * |
|  | Iba1^high^ CD32^high^ | 7.666 ± 2.082 | 19.69 ± 15.14 |  | 0.1049 | ns |
|  | Iba1^high^ CD32^low^ | 91.3 ± 2.425 | 73.85 ± 21.85 |  | 0.0830 | ns |
| CD163 | Iba1^low^ CD163^high^ | 0.5136 ± 0.5965 | 1.69 ± 2.535 |  | 0.7209 | ns |
|  | Iba1^high^ CD163^high^ | 3.686 ± 4.799 | 3.711 ± 4.842 |  | 0.6454 | ns |
|  | Iba1^high^ CD163^low^ | 95.48 ± 4.918 | 94.43 ± 7.357 |  | 0.5526 | ns |
| L-ferritin | Iba1^low^ L-Ferritin^high^ | 1.054 ± 1.439 | 16.23 ± 15.66 |  | 0.0006 | *** |
|  | Iba1^high^ L-Ferritin^low^ | 98.86 ± 1.534 | 83.33 ± 15.89 |  | 0.0006 | *** |


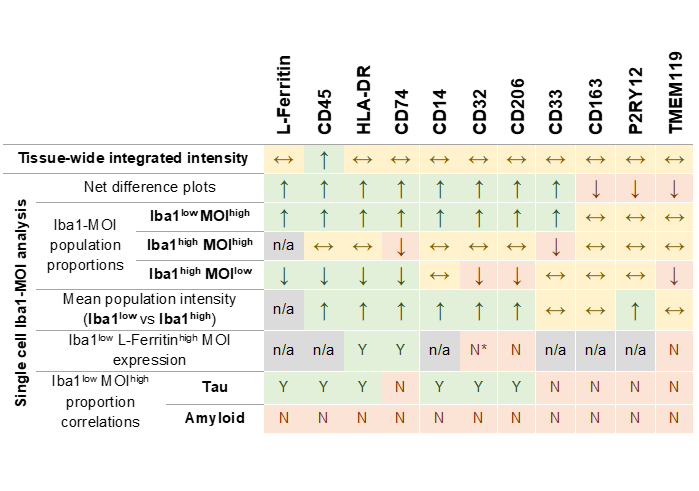


**Supplementary Figure 3: Summary of results presented in this study**


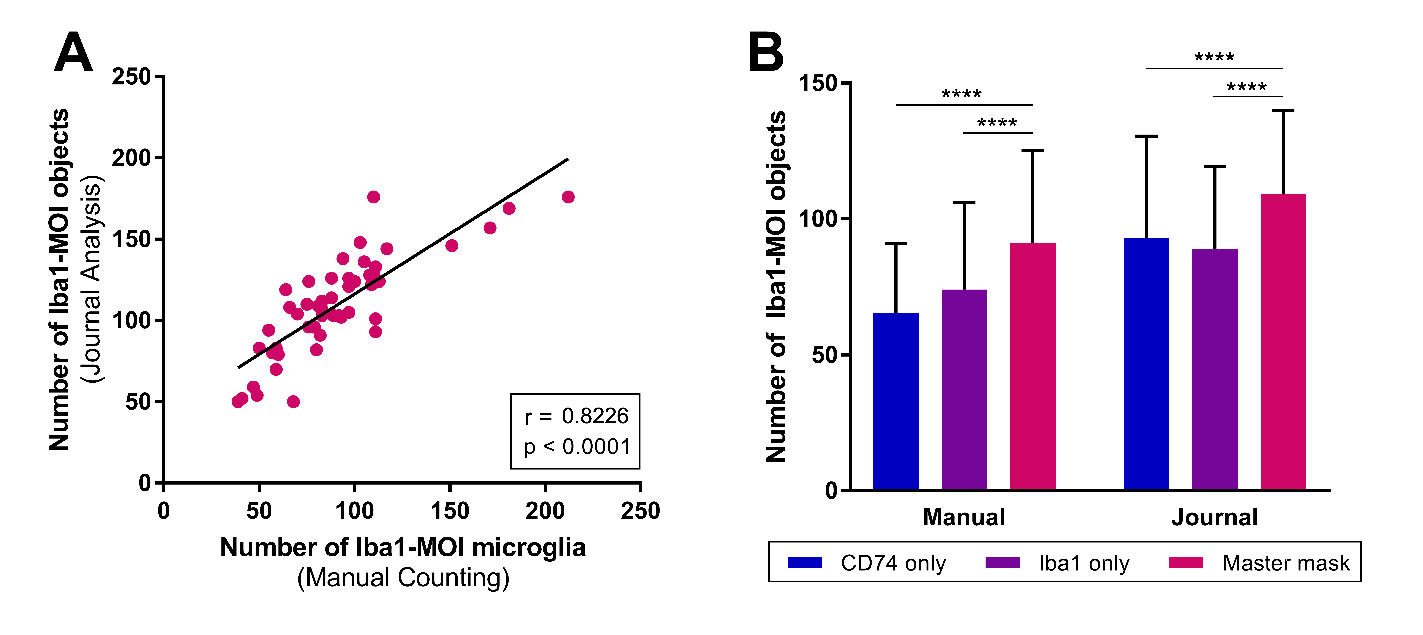


Supplementary Figure 4: Validation of the number of objects identified by MetaMorph automated analysis.

Significant positive linear correlation identified between the numbers of Iba1-MOI microglia manually counted in ImageJ and the number of Iba1-MOI objects identified by the MetaMorph journal’s master mask (A); data shown as a single point per 1500-by-1500-pixel image analysed (n = 52). Significantly more Iba1-MOI objects were identified by a master mask of both the Iba1 and MOI staining when quantified with the manual counting and automated analysis method; data shown as mean ± SD Iba1-MOI objects per 1500-by-1500-pixel image (n = 52).
